# Effects of mesenchymal stromal cells and human recombinant Nerve Growth Factor delivered by bioengineered human corneal lenticule on an innovative model of diabetic retinopathy

**DOI:** 10.3389/fendo.2024.1462043

**Published:** 2024-10-15

**Authors:** Letizia Pelusi, Jose Hurst, Nicola Detta, Caterina Pipino, Alessia Lamolinara, Gemma Conte, Rodolfo Mastropasqua, Marcello Allegretti, Nadia Di Pietrantonio, Tiziana Romeo, Mona El Zarif, Mario Nubile, Laura Guerricchio, Sveva Bollini, Assunta Pandolfi, Sven Schnichels, Domitilla Mandatori

**Affiliations:** ^1^ Department of Medicine and Aging Science, Center for Advanced Studies and Technology-CAST, University G. d’Annunzio of Chieti-Pescara, Chieti, Italy; ^2^ University Eye Hospital, Centre for Ophthalmology, University of Tübingen, Tübingen, Germany; ^3^ Dompé Farmaceutici SpA, Napoli, Italy; ^4^ Department of Medical, Oral and Biotechnological Sciences, Center for Advanced Studies and Technology-CAST, University G. d’Annunzio of Chieti-Pescara, Chieti, Italy; ^5^ Department of Neuroscience, Imaging and Clinical Sciences, University G. d’Annunzio of Chieti-Pescara, Chieti, Italy; ^6^ Dompé Farmaceutici SpA, L’Aquila, Italy; ^7^ Ophthalmology Clinic, Department of Medicine and Aging Science, “G. d’Annunzio” of Chieti-Pescara, Chieti, Italy; ^8^ Department of Experimental Medicine, University of Genova, Genova, Italy

**Keywords:** diabetic retinopathy, mesenchymal stromal cells, rhNGF, corneal lenticule, ocular delivery

## Abstract

**Introduction:**

Diabetic retinopathy (DR) is a microvascular complication of diabetes in which neurodegeneration has been recently identified as a driving force. In the last years, mesenchymal stromal cells (MSCs) and neurotrophins like Nerve Growth Factor (NGF), have garnered significant attention as innovative therapeutic approaches targeting DR-associated neurodegeneration. However, delivering neurotrophic factors directly in the eye remains a challenge. Hence, this study evaluated the effects of MSCs from human amniotic fluids (hAFSCs) and recombinant human NGF (rhNGF) delivered by human corneal lenticule (hCL) on a high glucose (HG) induced *ex vivo* model simulating the molecular mechanisms driving DR.

**Methods:**

Porcine neuroretinal explants exposed to HG (25 mM for four days) were used to mimic DR *ex vivo*. hCLs collected from donors undergoing refractive surgery were decellularized using 0.1% sodium dodecyl sulfate and then bioengineered with hAFSCs, microparticles loaded with rhNGF (rhNGF-PLGA-MPs), or both simultaneously. Immunofluorescence (IF) and scanning electron microscopy (SEM) analyses were performed to confirm the hCLs bioengineering process. To assess the effects of hAFSCs and rhNGF, bioengineered hCLs were co-cultured with HG-treated neuroretinal explants and following four days RT-PCR and cytokine array experiments for inflammatory, oxidative, apoptotic, angiogenic and retinal cells markers were performed.

**Results:**

Data revealed that HG-treated neuroretinal explants exhibit a characteristic DR-phenotype, including increased level of NF-kB, NOS2, NRF2 GFAP, VEGFA, Bax/Bcl2 ratio and decreased expression of TUBB3 and Rho. Then, the feasibility to bioengineer decellularized hCLs with hAFSCs and rhNGF was demonstrated. Interestingly, co-culturing hAFSCs- and rhNGF- bioengineered hCLs with HG-treated neuroretinal explants for four days significantly reduced the expression of inflammatory, oxidative, apoptotic, angiogenic and increased retinal markers.

**Conclusion:**

Overall, we found for the first time that hAFSCs and rhNGF were able to modulate the molecular mechanisms involved in DR and that bioengineered hCLs represents a promising ocular drug delivery system of hAFSCs and rhNGF for eye diseases treatment. In addition, results demonstrated that porcine neuroretinal explants treated with HG is a useful model to reproduce *ex vivo* the DR pathophysiology.

## 1 Introduction

Diabetic retinopathy (DR) is a leading cause of vision impairment and blindness in working-age populations worldwide (1). This is considered one of the most frequent complications of diabetes mellitus (DM) related to hyperglycemia and may occur in approximately 27% of patients with diabetes (2). Of note, high glucose concentration, a common characteristic of poorly controlled diabetes, plays a defining role in activating the mechanisms that cause DR, including genetic and epigenetic factors, inflammation, heightened production of free radicals, oxidative stress and vascular endothelial growth factor (VEGF), which induce vascular endothelial cell proliferation, migration, and increased vascular permeability (3–6).

Clinically, DR can be divided into two stages: non-proliferative (NPDR) and proliferative (PDR) (4). NPDR is asymptomatic and characterized by inflammation, loss of endothelial and neuronal cells, and retinal microvasculature abnormalities. As the disease progresses in PDR, neovascularization is characterized by the presence of fragile vessels, which can easily break and hemorrhage into the vitreous, leading to sudden vision loss and often retinal detachment (3).

Although for several years DR has been considered a microvascular complication of DM, recently growing evidence suggests that the vascular phase of DR might be preceded by a diabetes-driven neurodegenerative process, which particularly interests the retinal ganglion cells (RGC) (7). These findings led to the definition of DR as a highly specific neurovascular complication (8, 9).

Currently, in addition to controlling blood glucose, blood pressure, and serum cholesterol levels, various therapeutic approaches have become the standard for managing DR (10). These encompass laser photocoagulation, intravitreal injection of triamcinolone acetonide (IVTA) or anti-VEGF agents and intravitreal steroid implants (11). However, these options are principally used to target the late PDR stages, when vision is already significantly impaired (12). In addition, despite the discovery of therapeutic applications that have revolutionized the handling of DR, different side effects are related to its use, including endophthalmitis, intraocular inflammation, rhegmatogenous retinal detachment, tractional retinal detachment, intraocular pressure elevation, ocular hemorrhage, and ghost cell glaucoma (4). Thus, there is a strong need to develop novel treatments, particularly targeted at the initial phase of DR, where neurodegeneration is the driving force. From this perspective, there has been growing interest in stem cell (SCs) therapy and neurotrophins, with particular attention being paid to mesenchymal stromal cells (MSCs) owing to their modulatory potential. This is based on secreted soluble factors, including growth factors, cytokines, and chemokines, which are primarily responsible for therapeutic benefits through paracrine effects (13). Among the different cell sources, human SCs from amniotic fluid appear to be promising for the treatment of DR. Indeed, as reported by Costa et al., the secretome of fetal and perinatal human amniotic fluid SCs (hAFSCs) is enriched in immunomodulatory, anti-inflammatory, anti-fibrotic, and neurotrophic factors (14).

Neurotrophins, a family of structurally and functionally related growth factors, have gained attention for DR management because they are essential for the growth, differentiation, and survival of several cell types, including retinal neurons and glial cells. Among these, one of the main members is the nerve growth factor (NGF), which plays a key role in neurodegeneration, inflammation, vascular permeability, and injury processes involved in DR pathogenesis (15).

While numerous studies (16–21) have highlighted the positive effect of MSCs and NGF in *in vitro* and *in vivo* DR models, the delivery of these factors directly in the eye remains a challenge. Concerning this, we recently demonstrated the feasibility of using human corneal lenticules (hCLs), a thin and disc-shaped part of the cornea obtained and discarded during refractive surgery, as an ocular drug delivery system. In fact, we have proven that hCLs can be bioengineered with polylactic-co-glycolic-acid (PLGA) microparticles loaded with rhNGF (rhNGF-PLGA-MPs), and that active rhNGF is sustainably released from such bioengineered hCLs for up to 1 month (22). In addition, Gary Hin-Fai Yam emphasized the reinnervation potential of decellularized hCLs in grafted chick dorsal root ganglion models in the presence of NGF (23).

It is well established that tissue culture models have the advantage of preserving the different cell types within the tissue structure (24–26). However, to transfer experiments from animal models or animal tissues to humans, it is crucial to consider the anatomical, physiological, and morphological differences between species and humans when selecting a model organism (27). In this context, pigs are used as experimental model in various medical fields because of their anatomical and morphological similarities to humans (15, 28). In particular, the pig eye has a high morphological similarity to the human eye, especially in terms of size, retinal structure, and histology, making it suitable as a retinal DR model (27, 29–31).

Based on these assumptions, the present study aimed to evaluate the effects of hAFSCs and rhNGF, delivered by hCLs, on the molecular mechanisms underlying DR using an *ex vivo* model represented by porcine neuroretinal explants treated with high glucose (HG). In particular, by using the entire neuroretina, we can mimic the DR *milieu* across all retinal tissues cell types, enabling a thorough assessment of how HG treatment, as well as hAFSCs and rhNGF, could influence the entire retinal structure and its cellular components.

## 2 Materials and methods

### 2.1 Pig eyes handling, isolation and cultivation of retinal explants

Eyes were obtained from 6-month-old pigs euthanized by electrocution at a local abattoir and transported at 4°C to the laboratory within 3h of animal death. After arrival at the laboratory, the eyes were washed and disinfected prior to neuroretinal explant isolation. According to established procedure (32, 33), retinas were pierced with a dermal punch (∅ = 8 mm; Pmf medical AG, Germany) on the previously generated clover-leaf-like structures. These were placed in a petri dish with neurobasal medium (Neurobasal-A medium; Thermo Fisher Scientific, Karlsruhe, Germany) and neuroretinal explants were removed with a spoon and placed on a 12-well Millicell culture insert (Merck, Germany, with a pore size of 4 μm) containing 100 μL of retina culture medium per insert and 1 mL per well (50 mL Neurobasal-A medium supplemented with 2% B27 (Thermo Fisher Scientific, Karlsruhe, Germany), 1% N2 (Thermo Fisher Scientific, Karlsruhe, Germany), 1% penicillin/streptomycin (P/S), 0.5 μL ciliary neurotrophic factor (CNTF;Merck, Darmstadt, Germany), and 0.5 μL brain-derived neurotrophic factor (BDNF;Merck, Darmstadt, Germany) with the ganglion layer (GCL) facing up. Neuroretinal explants were placed in an incubator at 5% CO_2_ and 37°C for 3 h.

### 2.2 HG *ex vivo* model

3 h after explantation, the 8 mm neuroretinal explants were exposed to HG concentration for 96 h. To this end, retinal culture medium was replaced by a basal medium (1:1 retina culture medium and α-MEM with 10% of fetal bovine serum, Gibco-Life Technologies, Waltham, MA, USA, 1% L-glutamine and 1% penicillin/streptomycin (Sigma-Aldrich, St. Louis, MO, USA) added with D-glucose (HG-medium; SIGMA, St. Louis, MO, USA; G8270-100G) at a final concentration of 25 mM. After 48 h, HG medium was completely replaced with fresh medium. The same concentration of mannitol (25 mM) was added to 1:1 basal medium as the osmotic control.

### 2.3 hCLs collection and decellularization

The hCLs obtained from the cornea of healthy patients undergoing to small-incision lenticule extraction (SMILE) refractive surgical procedure were collected in accordance with the Declaration of Helsinki and following the “G. d’Annunzio” of Chieti-Pescara University’s Institutional Review Board and Ethical Committee approval (authorization n.: 03/07-02-2019). To obtain a human corneal keratocytes-free scaffold, hCLs were decellularized, as previously described (22). Briefly, the tissues were washed in phosphate buffer solution (PBS 1X, Sigma-Aldrich) and incubated in a 0.1% sodium dodecyl sulfate (SDS) solution for 24 h under 300 rpm agitation at room temperature (RT).

### 2.4 hAFSCs isolation

hAFSC were isolated from leftover samples of human amniotic fluid (hAF) collected by amniocentesis during II trimester prenatal diagnosis screening from the Fetal and Perinatal Medical and Surgery Unit and the Human Genetics Laboratory at IRCCS Istituto Gaslini hospital (Genova, Italy), following written informed consent and according to local ethical committee authorization (as obtained from Comitato Etico Territoriale Liguria, protocol P.R. 428REG2015). Donor’s age of AF ranged from 36 to 41 years, with an average age of 37.42 ± 0.32. hAF were processed as previously described (34–36) by centrifugation at 1200 rpm for 5 min to retrieve cellular components. The pellet was seeded on a glass coverslip in Chang Medium C (Irvine Scientific) supplemented with 1% L-glutamine and 1% penicillin/streptomycin (Gibco, Thermo Fisher Scientific) and cultured in an incubator at 37°C with a 5% CO_2_ and 20% O_2_ atmosphere. hAFSC were isolated from a population of adherent amniotic fluid mesenchymal stromal cells by immunomagnetic sorting for c-KIT expression (CD117 MicroBead Kit, Miltenyi Biotechnology). c-KIT+ hAFS were further expanded in Minimal Essential Medium (MEM)-alpha with 15% fetal bovine serum (FBS; Gibco - Thermo Fisher Scientific), 20% Chang Medium C (Irvine Scientific) with 1% L-glutamine, and 1% penicillin/streptomycin (Gibco - Thermo Fisher Scientific), in an incubator at 37°C with 5% CO_2_ and 20% O_2_ atmosphere. hAFSC were cultured and expanded to 3-4 passages before cryopreservation in liquid nitrogen until further application.

### 2.5 rhNGF-MPs preparation

PLGA microparticles (MPs) loaded with rhNGF were synthesized following a previously described customized double emulsion solvent evaporation technique (22). The MPs produced had an average size of 5 μm, and the encapsulation efficiency of rhNGF exceeded 65%.

### 2.6 hCLs bioengineering

Decellularized hCLs (Decell_hCL) were dehydrated for 2 h at 60°C and then bioengineered (BioE_hCL) in three different ways as follows:

with hAFSCs (BioE_hCL_A): Decell_hCLs were incubated for 72 h in a 96 well plate with 200 µL suspension of c-KIT^+^ hAFSCs (80 × 10^3^ cells/hCL; 5% CO_2_, 37°C).

with rhNGF-PLGA-MPs (BioE_hCL_B): rhNGF-PLGA-MPs were suspended in 0.175 mL of 0.9% NaCl (highest saturation degree). One Decell_hCL per tube was incubated with MPs solutions for 3h at RT with orbital shaking at 200 rpm. Following the incubation time, samples were finally washed 10 times in 0.4 ml of 0.9% NaCl. Fluo-PLGA-MPs (Cat. LGFG20K; Sigma-Aldrich). Louis, MO, USA) were used as controls for the bioengineering process with PLGA-MPs (BioE_hCL_Fluo-PLGA-MPs).

with hAFSCs and rhNGF-PLGA-MPs (BioE_hCL_C): For a double bioengineering, Decell_hCLs were first incubated with rhNGF-MPs solutions, as reported above. Subsequently, hCLs bio-engineered with rhNGF-MPs were incubated for 24 h with 200 µL of the hAFSCs suspension (80 × 10^3^ cells/hCL; 96 well plate).

All bioengineering processes were confirmed by immunofluorescence (IF) and scanning electron microscopy (SEM) analyses.

### 2.7 Immunofluorescence and histological analyses

For IF experiments neuroretinal samples were cryoprotected using Tissue Tek (Sakura, Umkirch, Germany) and frozen in liquid nitrogen. Neuroretinal explants were then cut on cryostat (10 µm sections) and fixed with 4% PFA. 1 h of 5% BSA blocking was performed. After washing, neuroretinal explants slices were stained with monoclonal Purified Mouse Anti-GFAP Cocktail (1:200; BD Pharmingen™, Franklin Lakes, New Jersey, USA, cat. 556330), Anti-Rhodopsin antibody (1:1000; Cambridge, UK, cat. ab98887), Anti-Nerve Growth Factor Receptor antibody (NGFR p75, 1:50; Sigma-Aldrich, St. Louis, MO, USA, cat. S-N5408), and Trka monoclonal primary antibody (1:50; Danvers, Massachusetts, USA cat. 2505S). Cy3 anti-mouse (1:200; Jackson ImmunoReasearch, Laboratories, cat. 111-165-144), Alexa Fluor 488 anti-rabbit (1:500; Invitrogen, Thermo Fisher Scientific, Waltham, MA, USA, cat. A11034) and Alexa Fluor 546 anti-mouse (1:500; Invitrogen, Thermo Fisher Scientific, Waltham, MA, USA, cat. A-11030) were used as the secondary antibodies. Counterstaining with DAPI (4′6′-diamidino-2-phenylindole; Thermo Fisher Scientific, Karlsruhe, Germany) was performed. The slices were mounted using FluorSafe (Merck, Darmstadt, Germany). Images were captured using a confocal microscope (Zeiss LSM-800; Carl Zeiss Meditec AG, Oberkochen, Germany). Staining intensity of GFAP and Rho were measured by using ImageJ software (NIH, United States ImageJ software, public domain available at: http://rsb.info.nih.gov/nih-image/).

To evaluate retinal tissue morphology, cryosections (10 μm) of control and HG-treated neuroretinal explants, were stained with Hematoxylin and Eosin (H&E). The sections were photographed under light microscopy objectives (10X and 20X) using the slide scanner system NanoZoomer. Quantification analyses was performed with Qu-Path 0.3.2 software using cell detection tool.

To confirm the bioengineering process, Decell_hCL and the three different BioE_hCLs were fixed in 4% paraformaldehyde (PFA; 10 min, RT) and blocked for 1 h at RT with 1% of albumin bovine serum (BSA) (SIGMA, St.Louis, MO, USA). Louis, MO, USA, A4503). hCLs were stained with paxillin (5 µg/mL: Thermo Fisher Scientific, Waltham, MA, USA, cat. MA5-13356) or phalloidin-rhodamine-conjugated primary antibody (1:300; Invitrogen, Thermo Fisher Scientific, Waltham, MA, USA, cat. R415). FITC-goat anti-mouse was used as secondary antibody (1:100; Jackson ImmunoResearch Europe Ltd., Cambridge House, St. Thomas’ Place, cat. 115-095-006). DAPI staining was used to determine the nuclei.

### 2.8 Scanning electron microscopy

Decell_hCLs and the three different BioE_hCLs were fixed and dehydrated using an ascending ethanol series (50%, 70%, and 100%), followed by immersion in hexamethyldisilazane and air drying at RT in a desiccator. The dried samples were then mounted onto SEM stubs and sputter-coated with gold layers (150 Å thick). Samples were examined using a Phenom XL microscope (Alfatest, Phenom World).

### 2.9 Co-culture of neuroretinal explants with BioE_hCL_Fluo-MPs

Control (CTRL) neuroretinal explants were placed in direct contact with BioE_hCL_Fluo-PLGA-MPs to assess the possible release of Fluo-PLGA-MPs from hCLs and their incorporation into the retinal layers. In detail, after four days of co-cultivation, BioE_hCL_Fluo-PLGA-MPs were removed from the co-culture system. Then, the neuroretinal explants were collected, cryoprotected and cut as described in 2.7 for the subsequent IF analyses performed with the Zeiss LSM-800 (Carl Zeiss Meditec AG, Oberkochen, Germany). Counterstaining with DAPI was performed to visualize retinal cells and layers.

### 2.10 Co-culture of HG-treated neuroretinal explants with BioE_hCLs

HG-treated neuroretinal explants were placed in direct contact with Decell_hCLs or with each of the three different BioE_hCLs to evaluate the effects of neurotrophic and regenerative factors. Specifically, hCLs were placed over the HG-treated neuroretinal explants. HG medium was completely replaced with fresh medium after 48 h of co-culture. After four days of co-cultivation, the BioE_hCL were removed from the co-culture system. Finally, neuroretinal explants were collected and processed for subsequent real-time PCR.

### 2.11 Real-time PCR

RNA isolation from neuroretinal explants was conducted using TRIzol reagent (Thermo Fisher Scientific, Waltham, MA, USA) according to previously established protocols (Pelusi L., 2022) following the manufacturer’s instructions. The quality and concentration of the total RNA were assessed using a NanoDrop 2000c spectrophotometer (Thermo Fisher Scientific, Waltham, MA, USA). A high-capacity cDNA Reverse Transcription Kit (Thermo Fisher Scientific, Waltham, MA, USA) was used for cDNA synthesis. The TaqMan Universal PCR Master Mix (Thermo Fisher Scientific, Waltham, MA, USA) and TaqMan Gene Expression Assay (Thermo Fisher Scientific, Waltham, MA, USA) probes for glyceraldehyde-3-phosphate dehydrogenase (GAPDH; Ss03375629_u1), glial fibrillar acid protein (GFAP; Ss03373547_m1), Cyclin Dependent Kinase Inhibitor 1A (p21; Ss06866662_m1), Nuclear factor erythroid 2-related factor 2 (NRF2; Ss06886078_m1), Nitric oxide synthase 2 (NOS2; Ss03374886_u1), Bcl2-associated X protein (Bax; Ss03375842_u1), B-Cell Leukemia/Lymphoma 2 (Bcl-2; Ss03375167_s1), nuclear factor kappa B subunit 1 (NF-kB1; Ss03388575_m1); Tubulin Beta 3 Class III (TUBB3; Ss06898264_g1) RHO, VEGFA (Ss03393990_m1) were used according to the manufacturer’s instructions. All samples were analyzed in duplicate. The relative expression of the target genes in each group was expressed as the fold change in mRNA expression by means of by 2^-ΔΔCt^ considering GAPDH as housekeeping.

### 2.12 Cytokine array

Conditioned media derived from HG-treated neuroretinal explants co-cultivated with each of the three BioE_hCLs were pulled (n ≥ 6) and analyzed using a semi-quantitative Cytokine Array (RayBiotech, Inc. Parkway Lane, GA, cat. AAP-CYT-1-8), according to the manufacturer’s instructions. After blocking the membranes, samples were pipetted into each well and incubated for 5 h at RT. After two washes, the biotinylated antibody cocktail was pipetted into each well and incubated overnight at 4°C. Afterwards, 1X HRP-Streptavidin was added and incubated for 2 h at RT. The immune complexes were visualized using the Detection Buffer (1:1 C+D) provided by the array kit. Data were processed using the UVITEC Alliance software, and spot signal intensities were quantified using the ImageJ software.

### 2.13 Statistical analyses

Statistical significance was analyzed using GraphPad Prism Software Analysis (version 9, San Diego, USA). D’Agostino and Pearson normality tests were performed. Then, data were analyzed via unpaired Student’s t-test or Mann-Whitney test for comparison between two groups. While, to compare more than two groups one-way ANOVA or Kruskal-Wallis followed by *post hoc* tests (Tukey or Dunn). Data are shown as the mean ± standard error (SEM). The value of *p* was set at 0.05.

## 3 Results

### 3.1 Effect of HG on porcine neuroretinal explants

**Figure 1:**
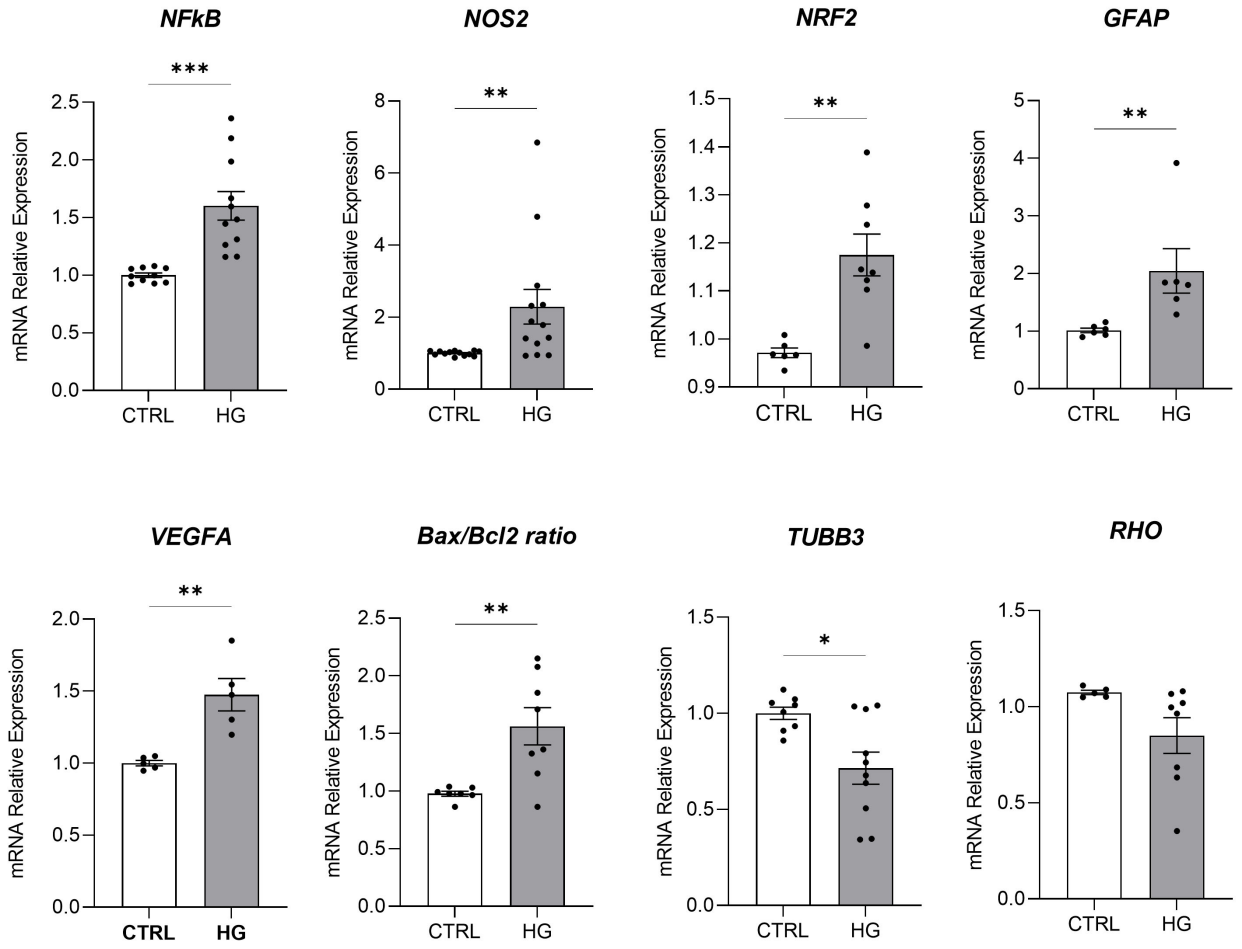
Molecular analyses of HG-treated porcine neuroretinal explants. mRNA levels of inflammatory/oxidative (*NFκB, NOS2, NRF2, and GFAP*), pro-angiogenic (*VEGFA*), apoptotic (*Bax/Bcl-2*), and retinal (*TUBB3* and *Rho*) markers in porcine neuroretinal explants cultured for 4 days in the presence or absence of HG (25 mM). Results are shown as the mean ± error standard (SEM) (*n*≥5); *p<0.05; **p<0.01; ***p<0.001.

**Figure 2:**
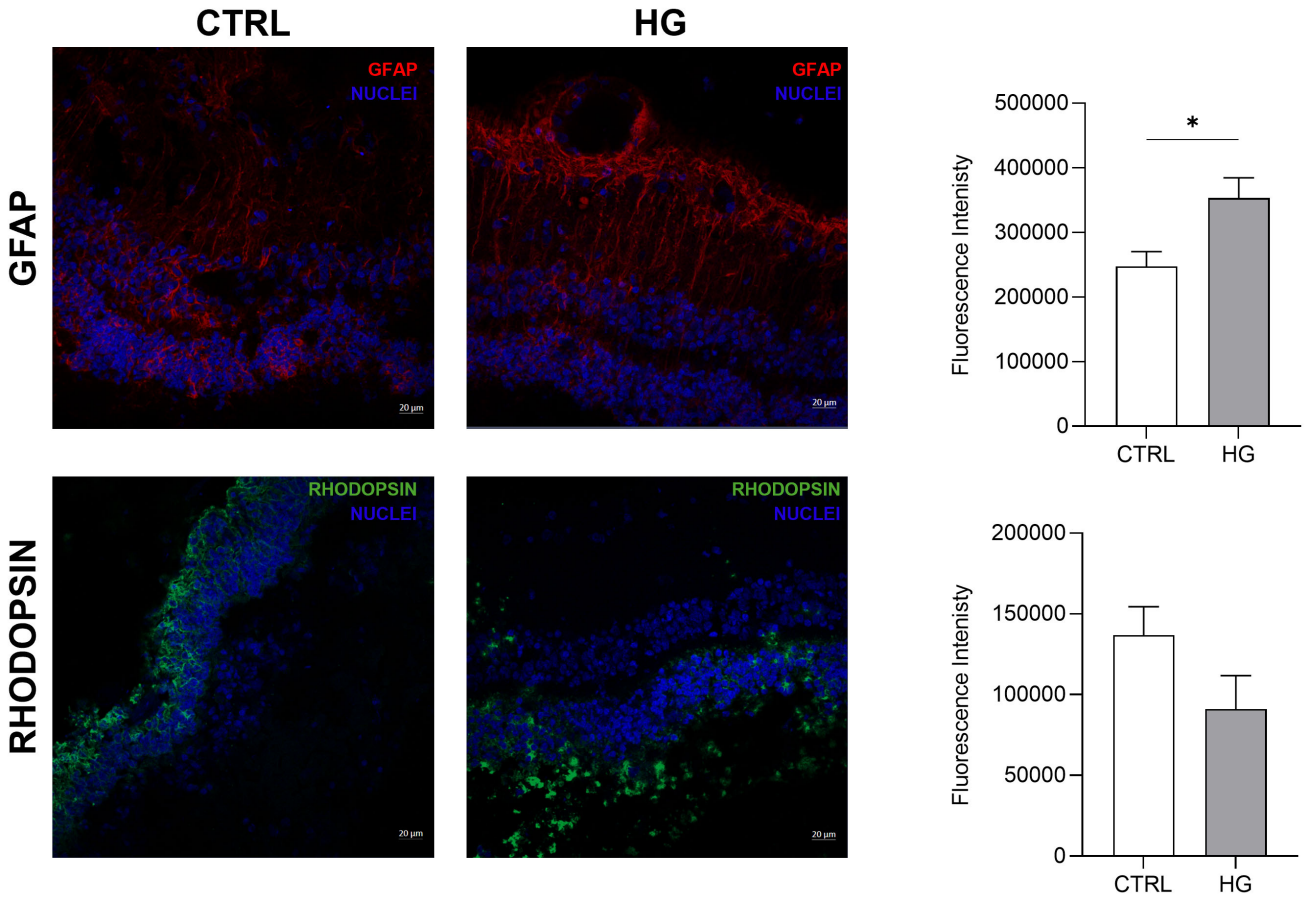
Immunofluorescence analyses of HG-treated porcine neuroretinal explants. Representative immunoflorescence (IF) images of CTRL and HG-treated neuroretinal explants and quantification of fluorescence intensity for GFAP (in red) and Rho (in green) markers. Nuclei were stained with DAPI (in blue) (n=3); *p<0.05.

**Figure 3:**
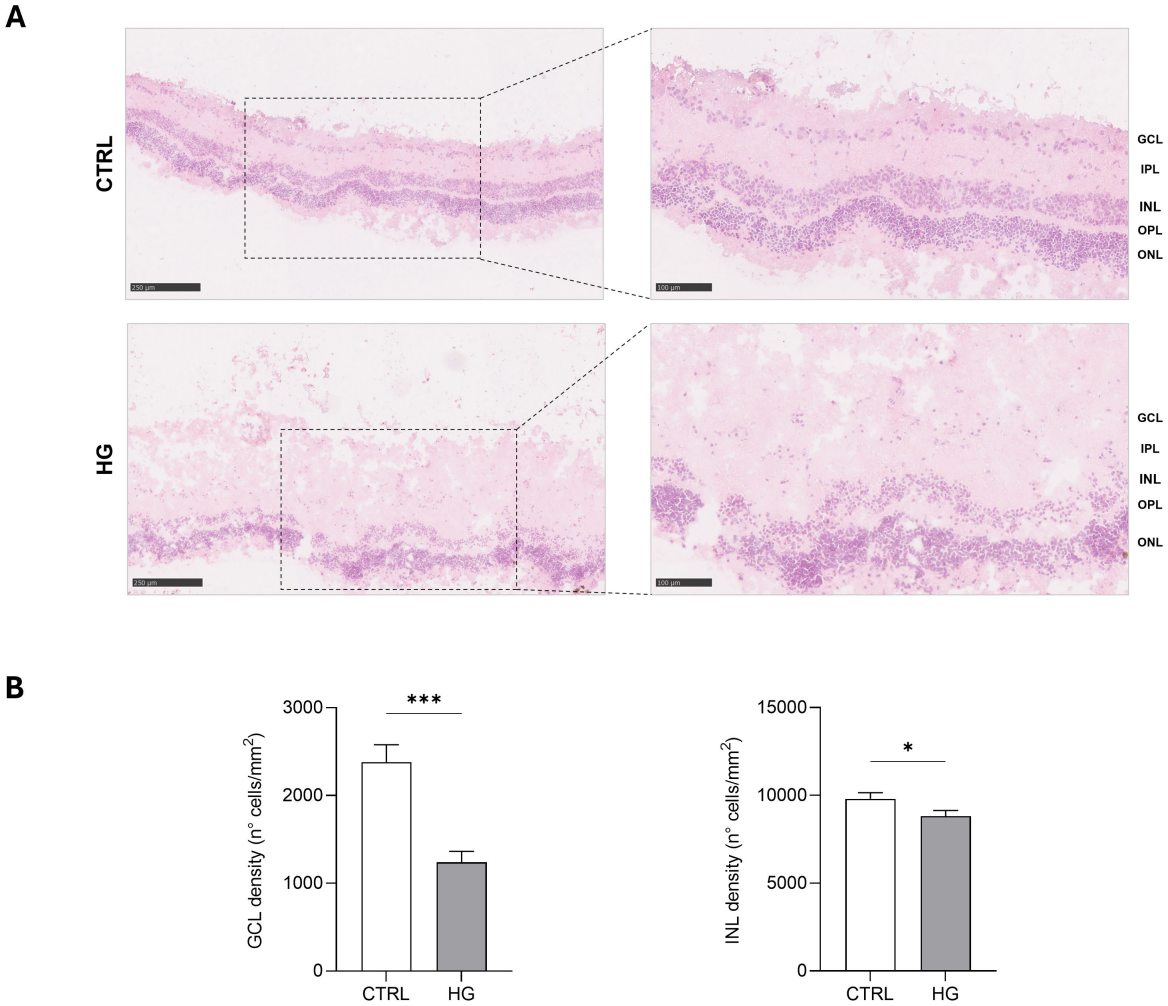
Hystological analyses of HG-treated porcine neuroretinal explants. **(A)** Representative H&E images (10X and 20X) of CTRL and HG-treated neuroretinal explants and **(B)** quantification anlyses of cells density expressed as number of cells/mm2 of area for GCL and INL (n=3); *p<0.05; ***p<0.001.

mRNA expression of markers associated to a pre-stage step of the DR-*ex vivo* model was evaluated to assess molecular changes associated to four days HG treatment (25 mM) in neuroretinal explants. Interestingly, HG treatment induced an upregulation of the inflammatory and oxidative stress processes, as shown by the significant increase of *NFkB*, *NOS2* and *NRF2*. In addition, *GFAP* expression was significantly increased by HG and as expected, this was accomplished with a significant enhance in *VEGFA*. Furthermore, induction of apoptosis was also observed following HG treatment, as evidenced by the notable increase in *BAX/Bcl-2*-ratio expression. This was accompanied by a significant decrease in the expression of *TUBB3* and a trend in downregulation of *RHO*, typical markers for retinal ganglion cells (RGCs) and photoreceptors respectively, indicating a reduction in their functionality (**Figure 1**). To validate the mRNA expression analyses, IF for GFAP and Rho protein expression were performed. As showed by the representative IF images and by the histograms in **Figure 2**, HG treatment induced a significant increase in GFAP expression, confirming the activation of an inflammatory process. While the reduction of Rho expression highlighted a retinal cells structure alteration (**Figure 2**). In support of this, the histological analyses of retinal morphology evaluated through H&E staining (**Figure 3A**) revealed tissue structure alterations in HG treated neuroretinal explants, primarily affecting the inner plexiform layer (IPL). Of note, the quantification analyses (**Figure 3B**) showed that HG treatment caused a significant reduction in the cells number of ganglion cell layer (GCL) and inner nuclear layer (INL). Finally, to confirm that the observed effects were specifically induced by HG treatment, neuroretinal explants were treated with mannitol (MANN; 25 mM) as an osmolarity control.

Treatment with mannitol did not induce any significant changes in the expression of *NOS2, NFkB, TUBB3*, and *GFAP*, thus confirming the specificity of the HG treatment, which was able to reproduce the DR scenario in *ex vivo* neuroretinal explants (**Supplementary Figure S1**).

### 3.2 Analyses of bioengineered hCLs

**Figure 4:**
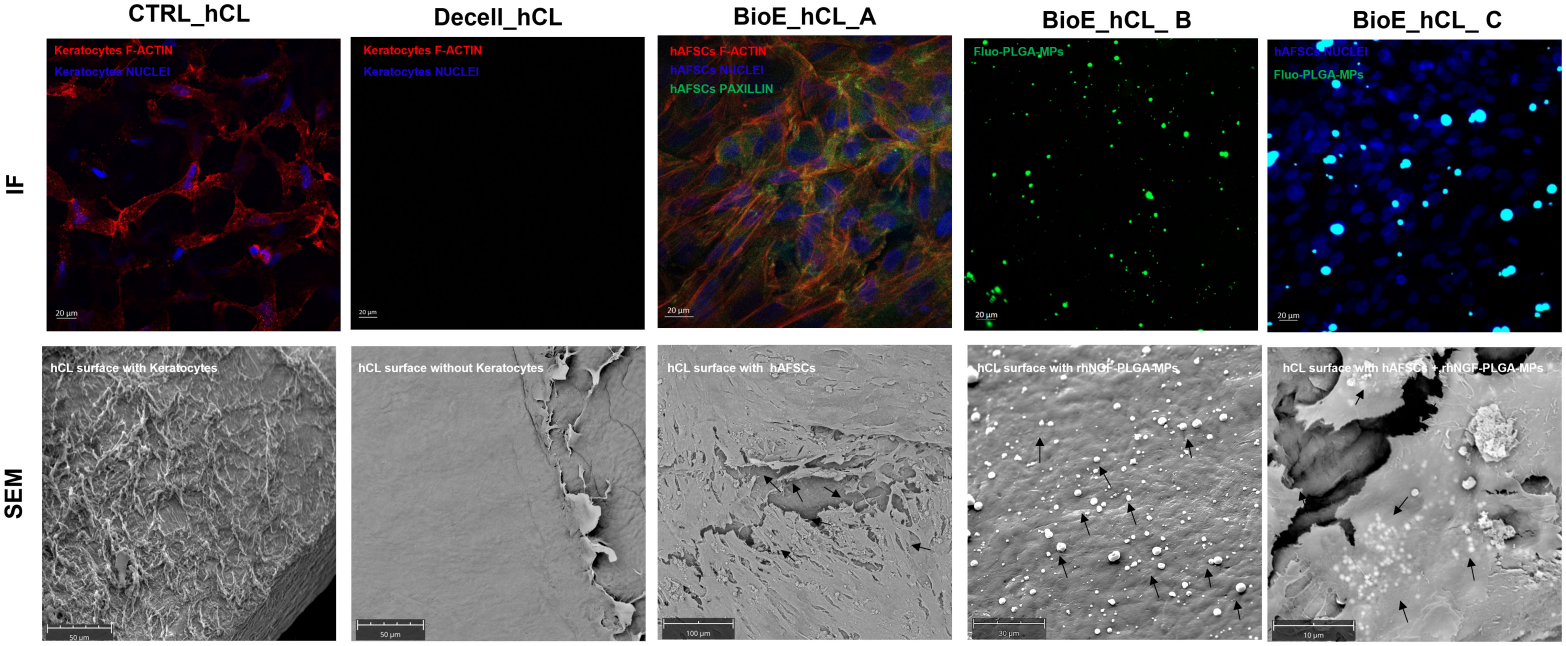
Analyses of BioE_hCLs. Representative immunoflorescence (IF) and scanning electron microscope (SEM) images: hCLs (CTRL_hCL); decellularized hCLs with SDS 0.1% (Decell_hCL); Decell_hCL bioengineered with hAFSCs (BioE_hCL_A); Decell_hCL bioengineered with Fluo-PLGA-MPs or rhNGF-PLGA-MPs (BioE_hCL-B); Decell_hCL bioengineered with hAFSCs and Fluo-PLGA-MPs or rhNGF-PLGA-MPs (BioE_hCL_C).

The bioengineering processes of hCLs were demonstrated using IF and SEM analyses. The efficacy of 0.1% SDS in removing from untreated control hCL (CTRL_hCL) both cellular and nuclear material derived from human corneal keratocytes, was firstly confirmed through the IF staining of Decell_hCLs for phalloidin (intracellular F-actin) and DAPI (nucleic acids) (**Figure 4**, Decell_hCL image in IF panel). This result was corroborated by the SEM images which showed how the surface of Decell_hCLs (Figure, Decell_hCL image in SEM panel) appears smooth when compared to the surface of untreated control hCLs (**Figure 4**, CTRL_hCL image in SEM panel). Subsequently, the feasibility of bioengineering hCLs with hAFSCs (BioE_hCL_A condition) was proven. To this end, Decell_hCLs were incubated for 72 h with hAFSCs and then IF staining for the nuclei and F-actin revealed the ability of hAFSCs cells to repopulate the surface of Decell_hCLs (**Figure 4**, BioE_hCL_A image in IF panel). Of note, the presence of focal adhesions of hAFSCs was also found as evidenced by the presence of paxillin localized along the cell periphery and interacting with F-actin on the plasma membrane (**Figure 3**, BioE_hCL_A image in IF panel). In support of this, SEM images clearly displayed the monolayer of hAFSCs and their close adherence on Decell_hCL surface (**Figure 4**, BioE_hCL_A image in SEM panel). Then, the BioE_hCL_B condition was firstly verified in IF incubating Decell_hCLs with a suspension of Fluo-PLGA-MPs, which were used as bioengineering process control. In detail, confirming our previous findings (22), the IF images confirmed that Fluo-PLGA-MPs are able to attach the hCLs surface (**Figure 4**, BioE_hCL_B image in IF panel). While the bioengineering process with rhNGF-PLGA-MPs was confirmed by SEM analyses which highlighted the attachment of rhNGF-PLGA-MPs to the collagen fibers of Decell_hCLs surface (**Figure 4**, BioE_hCL_B image in SEM panel).

Finally, the feasibility of double-bioenginering Decell_hCLs with hAFSCs and rhNGF-PLGA-MPs was investigated. In particular, for IF analyses, Decell_hCLs were first incubated with Fluo-PLGA-MPs solutions and then with hAFSCs. Of note, IF images demonstrated that both Fluo-PLGA-MPs and hAFSCs are simultaneously able to adhere and attach hCLs surface (**Figure 4**, BioE_hCL_C image in IF panel). These results were validated by SEM analyses which showed the presence of rhNGF-PLGA-MPs below the hAFSCs monolayer in the doubly bioengineered hCL thus demonstrating for the first time the successful development of a doubly bioengineered hCL (**Figure 4**, BioE_hCL_C image in SEM panel).

### 3.3 Expression of NGF receptor and release of Fluo-MPs from BioE_hCLs in the neuroretinal explant

Before assessing the effect of BioE_hCLs on our *ex vivo* model, we evaluated the expression of NGF receptors (NGFR p75 and TrkA) in the neuroretinal explants and the possible release of Fluo-PLGA-MPs from BioE_hCLs in the retina layers.

**Figure 5:**
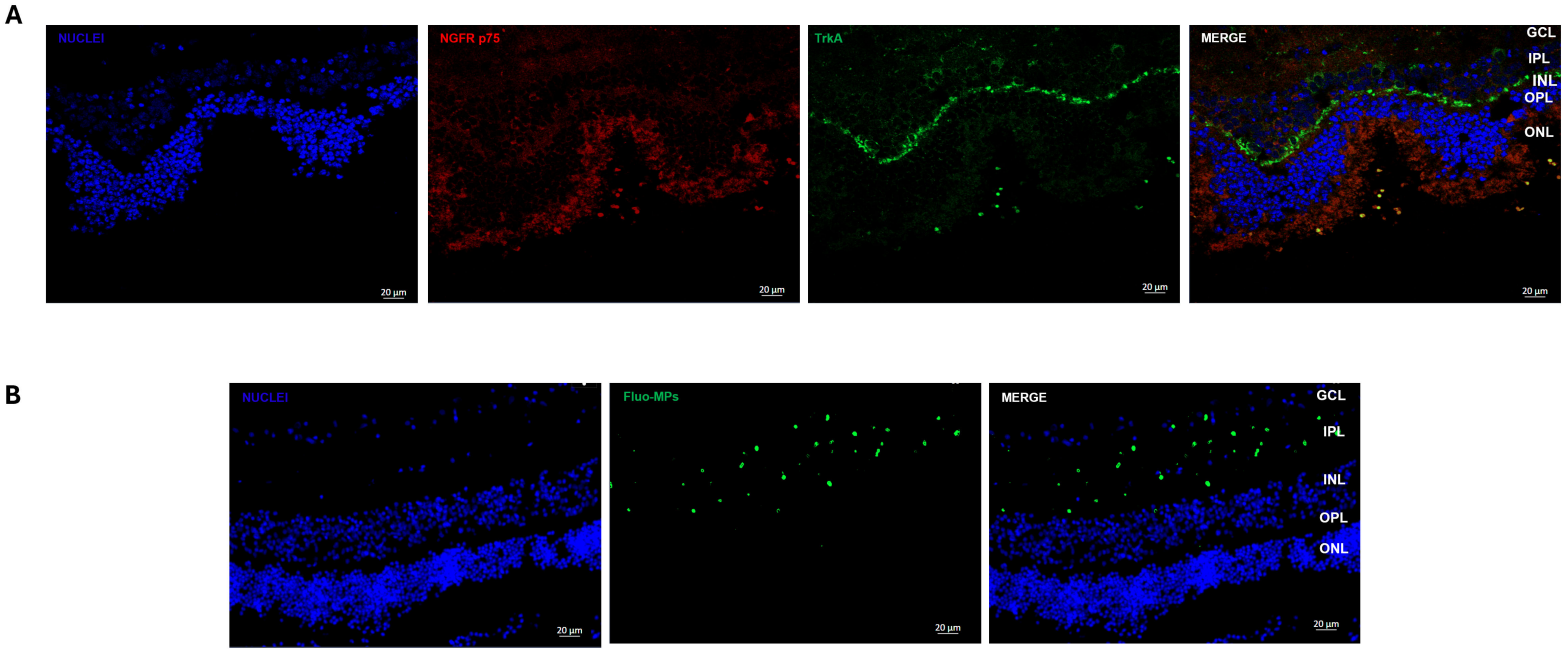
Immunofluorescence analyses of NGF receptor and Fluo-MPs in porcine neuroretinal explants. Representative immunofluorescence (IF) images of **(A)** NGF receptors in porcine neuroretinal explants. Alexa Fluor 576 was used to stain NGFR p75 and Alexa Fluor 488 for TrkA, and **(B)** Fluo-PLGA-MPs were incorporated into the porcine neuroretina layers after release from BioE_hCL.

Regarding NGF receptor expression, representative IF images revealed that both NGF receptors are expressed in porcine retinal cells. In particular, TrkA was highly expressed in the Inner Plexiform Layer (IPL), whereas NGFR p75 was expressed in the Outer Nuclear Layer (ONL) (**Figure 5A**).

Our previous results (22) demonstrated that hCL bioengineered with rhNGF-PLGA-MPs sustained the release of active rhNGF for up to one month. Hence here we investigated whether the growth factors and MPs, probably released from BioE_hCL, were able to reach the neuroretinal explants. This was achieved by using Fluo-PLGA-MPs as a control delivery and thus co-culturing the neuroretinal explants with BioE_hCLs_Fluo-PLGA-MPs.

Representative IF images (**Figure 5B**) showed that Fluo-PLGA-MPs were released from BioE_hCLs and subsequently incorporated into the retinal layers, particularly into theIPL.

### 3.4 Effect of hAFSCs and rhNGF released from BioE_hCLs on the *ex vivo* DR model: mRNA expression analyses

**Figure 6:**
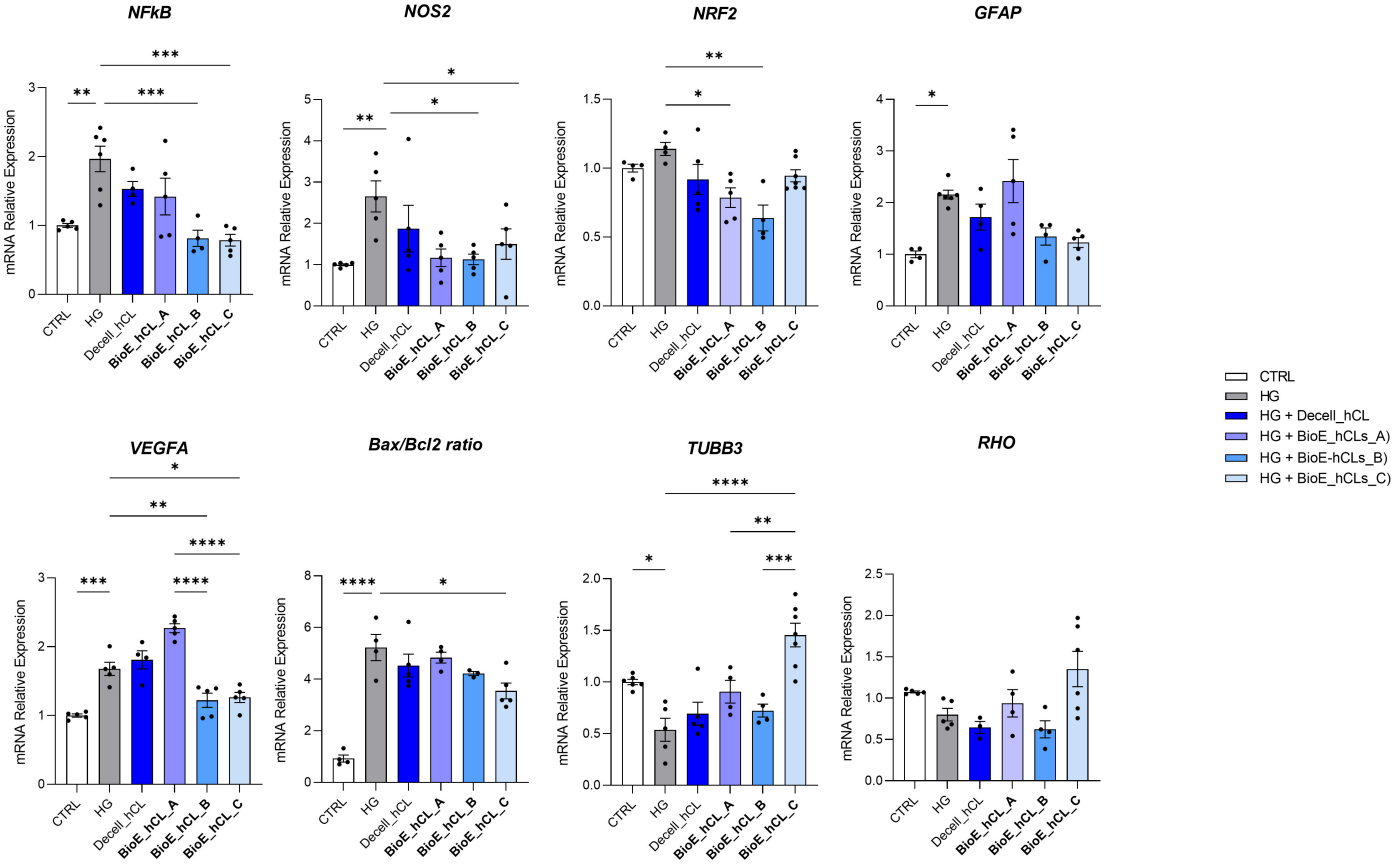
Molecular analyses of HG-treated porcine neuroretinal explants co-cultured with BioE_hCL. mRNA levels of inflammatory/oxidative (*NF-κB, NOS2, NRF2, and GFAP*), pro-angiogenic (*VEGFA*), apoptotic (*Bax/Bcl-2*), and retinal (*TUBB3* and *Rho*) markers in HG-treated porcine neuroretinal explants co-cultured with each of the three BioE_hCL. Results are shown as the mean ± error standard (SEM) (*n*≥4); *p<0.05; **p<0.01; ***p<0.001; ****p<0.0001.

HG-treated neuroretinal explants were placed in direct contact with BioE_hCL for four days. Interestingly, depending on the markers analyzed, we observed different effects after co-culturing with each of the three BioE_hCL (**Figure 6**).

Regarding *NFKb, NOS2, NRF2*, and *Bax/Bcl-2*, the co-culture with each of the three BioE_hCLs reduced their expression. However, statistical significance was only achieved with BioE_hCLs_B and BioE_hCLs_C, suggesting that the inflammatory and oxidative stress processes induced by HG treatment may be mainly modulated by the release of rhNGF.

The co-culture with BioE_hCL_B and BioE_hCL_C also reduced the expression of *GFAP* in the HG-treated neuroretinal explant. On the contrary, *GFAP* levels were improved following the co-culture with BioE_hCL_A. This condition also significantly increased *VEGFA* expression, probably due to the release of pro-angiogenic factors from hAFSCs. Of note, this pro-angiogenic effect was significantly reverted in the BioE_hCL_B and BioE_hCL_C conditions compared to the HG and BioE_hCL_ A ones.

Regarding the retinal cell’s markers, a similar pattern was observed for *TUBB3* and *RHO*. Although no difference was found in BioE_hCL_A and BioE_hCL_B compared to HG treatment, the reduction of *TUBB3* and *RHO* induced by HG treatment was significantly reversed in BioE_hCL_C, indicating a synergistic effect between rhNGF and hAFSCs in the double bioengineered hCL.

To better appreciate this synergic effect on our DR*-ex vivo* model, we normalized the computed values of BioE_hCL_C versus the HG condition (**Table 1**). Notably, this comparison highlighted that co-culture with BioE_hCL_C significantly reduced inflammatory (*NFkB, NOS2, GFAP*), oxidative (*NRF2*), angiogenic (*VEGF*), apoptotic (*Bax/Bcl-2*), and retinal cell (*TUBB3 and RHO*) markers, reinforcing the assumption that hAFSCs and rhNGF have a synergistic effect.

**Table 1:** HG-treated porcine neuroretinal explants co-cultured with BioE_hCL_C.

Markers	HG	BioE_hCL_C	*p*-value	Sig. level
*NFkB*	1.00 ± 0.00	0.41 ± 0.099	0.0001	***
*NOS2*	1.00 ± 0.00	0.59 ± 0.32	0.006	**
*NRF2*	1.00 ± 0.00	0.77 ± 0.1	0.03	*
*GFAP*	1.00 ± 0.00	0.57 ± 0.99	0.02	*
*VEGFA*	1.00 ± 0.00	0.76 ± 0.099	0.0026	**
*Bax/Bcl2*	1.00 ± 0.00	0.69 ± 0.13	0.0002	***
*TUBB3*	1.00 ± 0.00	3.017 ± 0.62	<0.0001	****
*RHO*	1.00 ± 0.00	1.72 ± 0.66	0.004	*

Normalized computed values of BioE_hCL_C versus HG condition. *p<0.05; **p<0.01; ***p<0.001; ****p<0.0001.

### 3.5 Effect of hAFSCs and rhNGF released from BioE_hCLs on the *ex vivo* HG-model: cytokine release analyses

To strengthen the mRNA expression results, conditioned media collected from each experimental condition were analyzed using a 48-cytokine array.

**Figure 7:**
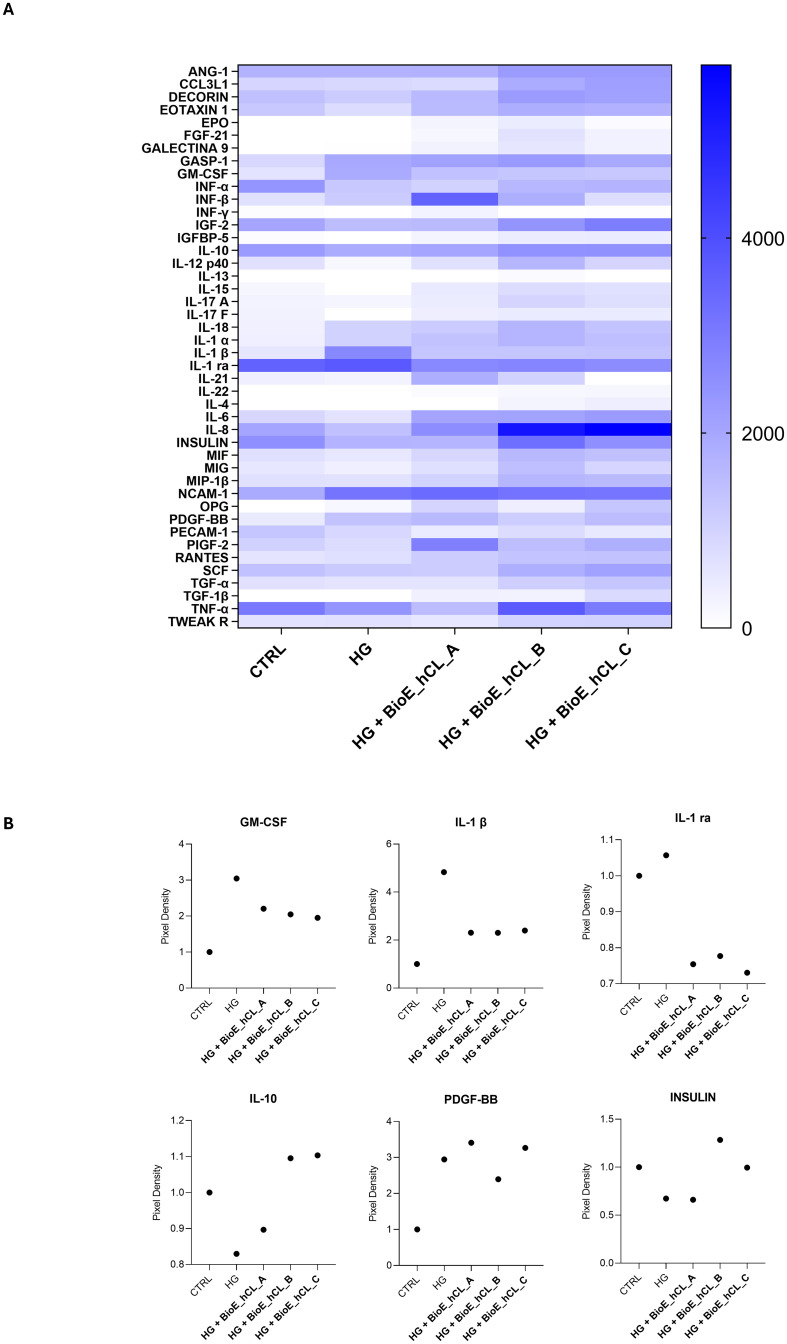
Cytokine array analyses of HG-treated porcine neuroretinal explants co-cultured with BioE_hCL. Cytokine release **(A)** Heatmap and **(B)** scatter dot plot showing the release of pro-inflammatory (GM-CSF, IL-1β, and IL-1 ra), anti-inflammatory (IL-10), pro-angiogenic (PDGF-BB), and neuroprotective (INSULIN) cytokines in conditioned media of CTRL and HG-treated porcine neuroretinal explants co-cultured with each of the three BioE_hCL. (n ≥ 6 for pooled conditioned media).

In general, as reported by the heatmap in **Figure 7A**, a modulation of cytokines involved in inflammation, angiogenesis, and cell growth was observed. Notably, the values of the principal markers of interest were extrapolated and plotted.

As expected, the dot plots shown in **Figure 7B** disclosed that HG treatment activated an inflammatory process in the neuroretina explants, as revealed by the increased release of pro-inflammatory cytokines such as granulocyte-macrophage colony-stimulating factor (GM-CSF), interleukin 1β (IL-1β), and interleukin 1 Receptor Antagonist (IL-1 ra) and by the decrease of the anti-inflammatory cytokine IL-10.

Notably, the release of pro-inflammatory cytokines was reduced in HG-treated neuroretinal explants co-cultured with each of the three BioE_hCLs. In contrast, IL-10 levels increased only in BioE_hCL_B and BioE_hCL_C. In addition, HG induced an increase in platelet-derived growth factor-BB (PDGF-BB), indicating activation of the neovascularization process. This was successively reversed in the HG-*ex vivo* neuroretinal explants treated with BioE_hCL_B and BioE_hCL_C. Interestingly, we also observed a decrease in INSULIN levels in HG-treated neuroretinal explants, confirming that HG reduced the levels of this important neuroprotective factor (37). Notably, co-culture with BioE_hCL_B and BioE_hCL_C restored INSULIN levels.

## 4 Discussion

DRis a complex ocular disease affecting a high percentage of diabetic patients (38). Even though it is considered a microvascular complication of DM, recent studies define DR as a tissue-specific neurovascular impairment of multiple retinal cells, including neurons, glial cells, and vascular cells that constitute the neurovascular units (NVUs) (8, 39). Therefore, the development of new therapeutic approaches for the management of DR associated neurodegeneration requires in depth investigation of the underlying molecular mechanisms (40).

In this context, several *in vitro* and *in vivo* experimental models of DR have been developed over the years which, however, are affected by different drawbacks (40–44). Cell culture models fail to replicate *in vivo* conditions because they lack a tissue microenvironment, which can significantly influence cellular responses. Likewise, *in vivo* models encounter growing ethical and regulatory challenges in the animals use (25). Therefore, tissue culture models have been highlighted as promising alternatives, especially in the field of ocular disease such as DR (25, 27, 45–48). However, although the anatomy of pig eye is highly similar to that of the human eye (29), no studies have explored the feasibility of replicating the DR *milieu* in porcine neuroretinal explants.

In light of this, in the present study, porcine neuroretinal explants were collected and treated with HG (25 mM), allowing us to evaluate the effects of HG in all retinal cell types thus replicating the diabetic *milieu* on the entire retinal structure. Notably, HG treated neuroretinal explants showed elevated expression of *NFκB*, implying the onset of a pro-inflammatory state, the initiation of a pro-apoptotic program leading to DNA damage through the increase of reactive oxygen species (ROS) production (49, 50). Additionally, in our *ex vivo* model, HG caused an increase in the *Bax/Bcl-2* ratio and *Nrf2* levels, supporting the relationship between NFκB activation and cell apoptosis. Indeed, it is known that both factors work together to maintain redox homeostasis in healthy cells and this regulation may be disrupted under DR condition (51–56).

Among the pro-inflammatory and oxidative stress mediators involved in the early stage of DR, NOS2 plays a critical role in tissue injury, neurodegeneration, and cell apoptosis. According to the state of the art (57–60), we found a significant increase of the *NOS2* level following the HG treatment.

Moreover, in line with the study of Lechner and colleagues (61), our DR model also underlined the activation of a neurodegenerative process revealed by the GFAP modulation, confirmed also by the IF analyses performed on cryosections of control and HG-treated neuroretinal samples.

Furthermore, although we also observed a modulation of the vascular markers VEGF, we were unable to evaluate the microvascular changes typical of DR. Indeed, in our model, the porcine neuroretinal explants were treated with HG for four days, which is a relatively short period and likely insufficient to observe significant microvascular changes. Furthermore, despite the porcine eye being represents an excellent research model, largely due to its anatomical and vascular similarities to the human eye (29), there are important differences that must be acknowledged. Unlike humans, where the central retinal artery and vein travel within the optic nerve before reaching the optic disc, pigs have retinal vessels that originate from chorioretinal arteries and veins at the optic disc periphery (62). For these reasons and since from the start of the enucleation process there is probably a rapid degeneration of vascular components, principally due to the lack of blood flow and biomechanical tissue support, it was difficult to study microvascular changes in our model, shifting the focus mainly to neural damage.

In addition to the markers discussed above, a decreased expression of *TUBB3* and *RHO* was found confirming that, RGCs and photoreceptors respectively, were affected to the HG treatment (63). In support of this, in line with Jeong JS and colleagues (64) H&E staining also revealed evident morphological changes in HG treated neuroretinal explants which mainly interest the IPL. Interestingly, compared to the CTRL group, a reduction in the numbers of nuclei in the GCL and INL were observed in HG treated samples, thus supporting the gene expression data.

Despite these observations, to note is that just the severing of the optic nerve initiates a degenerative program which primarily affect the RGCs (27, 65). This could influence the interpretation of RGC state, representing a limit of our model. However, although the model cannot fully discriminate the effects of HG treatment from the impact of optic nerve severing due to the enucleation process, the observed differences between the CTRL neuroretinal explants (basal condition) and the HG treated ones suggest that HG significantly contributes to worsen retinal damage.Considering the neurodegenerative signature of DR, recently gained attention has been focused on the development of new therapeutic approaches based on the use of MSCs and the replacement of neurotrophic factors (15).

MSCs therapy has been recognized as one of the most effective treatments for various degenerative diseases, including DR conditions (66, 67). It is well established that MSCs release several factors, such as insulin-like growth factor (IGF), VEGF, basic fibroblast growth factor (bFGF), BDNF, ciliary neurotrophic factor (CNTF), and NGF, which exert anti-apoptotic, angiogenic, pro-regenerative, and anti-fibrotic effects. Several studies demonstrated that intravitreal injections of different sources of MSCs promote in DR models the downregulation of GFAP, IL-1β, TNF-α, IL-10, and VEGF protein expression (18, 68, 69). While for the use of neurotrophic factors, experimental evidence has proven the effectiveness of NGF treatment in degenerative diseases of the retina, such as glaucoma, retinitis pigmentosa, and diabetic retinopathy (70–72). However, the direct delivery of these factors to the eye remains a challenge.

As regard of this, we previously demonstrated the feasibility of using hCLs as a natural ocular drug delivery system able of releasing rhNGF-PLGA-MPs for one month (22). In particular, Decell_hCL, through a phenomenon of ‘soaking” allows the rhNGF-PLGA-MPs to adhere uniformly to the hCL surface and incorporated within its stroma. Consequently, rhNGF-PLGA-MPs do not form covalent bonds but rather adhere to the hCL surface due to the polymeric and mucoadhesive properties of PLGA, which facilitate interaction with the collagen fibers of the hCLs. Thus, the mucoadhesive property of PLGA resulted particularly advantageous as allows to rhNGF-PLGA-MPs to adhere to the collagen fibers on the surface of the hCL, preventing them from falling off even after the washing phase of the procedure also allowing the sustained release over time of rhNGF. Furthermore, recently it has also been shown that hCLs can be recellularized with primary human stromal fibroblasts or with MSCs (73–75). In the wake of these studies, we demonstrated the feasibility of bioengineering hCLs with both hAFSCs and rhNGF-PLGA-MPs and their effect on the *ex vivo* DR model. Interestingly, by co-culturing each of the three BioE_hCLs here developed with HG-treated porcine neuroretina, we observed that hAFSCs and rhNGF ameliorate the HG induced damaged status. Nevertheless, a closer investigation showed that depending on the markers analyzed, the three BioE_hCLs displayed different patterns.

Among the existing sources, we selected hAFSCs thanks to their secretome enriched in proregenerative and neuroprotective factors (14). In detail, we found that hCLs bioengineered with only hAFSCs (BioE_hCLs_A) did not restore the increased levels of *GFAP* and *VEGF* in the HG-treated neuroretinal explant. While it reduced HG induced inflammation, oxidative stress, and apoptosis probably due to the release of well-known anti-inflammatory, immunomodulatory, and neurotrophic factors present in the hAFSCs secretome. Therefore, assuming that the effects are mediated by the release of paracrine factors from hAFSCs, it would be interesting to evaluate in the future the possibility of bioengineering hCLs with EVs derived from hAFSCs, thereby overcoming the current limitations in the clinical application of these cells.

As regard the replacement of neurotrophic factors, it was found that in DR rats model the retrobulbar injection of NGF attenuates visual damage by reducing RGCs apoptosis (20). However, ST-induced diabetic rats treated with NGF eye drops showed a non-significant trend toward protecting ganglion cells from diabetes-induced degeneration (76, 77). Interestingly, our data strikingly concurs with these results since the rhNGF released from BioE_hCLs_B significantly decreased inflammation, oxidative stress, neovascularization, and apoptosis. Probably, the rhNGF binding to its high affinity TrkA receptor, activating pathways like Ras-MAPK, ERK, PI3K-Akt, and PLC-ϒ (78–80). However, rhNGF did not improve the levels of retinal markers such as *TUBB3* and *RHO*.

To confirm that these results were due to the release of rhNGF from BioE_hCLs, we co-cultured neuroretinal samples with BioE_hCL_Fluo_PLGA-MPs, as delivery control. Interestingly, we provided evidence that Fluo-PLGA-MPs were released by BioE_hCL_Fluo-PLGA-MPs and that these were internalized into the retinal layer. This confirmed our previous results (22) and allowed us to hypothesize that BioE_hCLs were also able to release hAFSC-derived paracrine factors.

Although various studies have investigated the effects of MSCs and NGF on DR-model, none have explored the potential synergistic effect that could arise from their simultaneous release. Here, for the first time, we propose that they might have synergistic beneficial effects. Indeed, when BioE_hCLs_C were co-cultured with HG-treated neuroretinal explants a better effect was displayed compared to BioE_hCLs_A and BioE_hCLs_B, since a significant decrease of *NFkB*, *NOS2*, *VEGFA*, and *Bax/Bcl2* and an increased in *TUBB3* expression was observed. Additionally, a trend in the downregulation of *Nrf2* and *GFAP* and in the upregulation of RHO were detected. In accordance with Zha et al. (81) we hypothesis that the rhNGF released stimulates hAFSCs paracrine factor production through the TrkA-MSCs receptor interaction thus resulting in a synergistic effect.

Although the results obtained are promising, the study here reported has some limitations. Firstly we considered a sample size of neuroretinal explants equal to or greater than five (n ≥ 5) external factors such as the time of animal euthanasia as per day or night, which may affect photoreceptor activation, as well as the variability among animals often make it difficult to achieve statistical significance. Furthermore, the present study mainly focused on profiling the modulation of mRNA expression. A significant technical challenge we encountered was performing IF or histological analyses on neuroretinal explants following co-culture with BioE_hCLs. Indeed, due to the close contact between BioE_hCLs and the retinal explants, removing the BioE_hCLs could potentially cause a damage in the neuroretinal tissue structure, making the protein markers analyses performed directly *ex vivo* on the retina difficult. However, to support our mRNA expression results, we analyzed the conditioned medium of neuroretinal explants co-cultured with BioE_hCLs to quantify the levels of 48 cytokines involved in the processes of inflammation, oxidative stress and neovascularization. This evidenced the reduced release of GM-CSF, IL-1β, IL-1ra, and PDGF-BB, along with the increased levels of IL-10 and INSULIN. An additional limit is the difficulty in maintaining neuroretinal explants in *ex vivo* culture for extended periods, as they tend to undergo spontaneous degeneration through programmed cell death, including apoptosis and necroptosis (82). This did not allow us to observe the long-term effects of HG, especially the factors released by BioE_hCLs.

Nevertheless overall, our results are encouraging and provide compelling evidence that hAFSCs and rhNGF can modulate the molecular mechanisms underlying DR following their release from BioE_hCLs, which might be proposed as a valuable ocular delivery system. Notably, we also successfully demonstrated the feasibility of mimicking DR *ex vivo* by treating porcine neuroretinal explants with HG concentrations, thereby replicating the disease’s *milieu*. This innovative approach not only sets the stage for more detailed studies but also paves the way for transitioning to an *in vivo* experimental model that will enable us to confirm the effects of hAFSCs and rhNGF on DR. Furthermore, the employment of an *in vivo* experimental model will allow us to identify the optimal implantation site for BioE_hCL, ensuring sufficient exposure and release of neurotrophic and regenerative factors to the retina providing us a better guidance on the subsequent treatment planning to move forward to possible clinical studies. Indeed, this is a proof-of-concept study that does not currently support the feasibility and efficacy of this transplant approach in DR patients. Therefore, further studies will be needed to assess its potential for clinical application.

## Data Availability

The original contributions presented in the study are included in the article/**Supplementary Material**. Further inquiries can be directed to the corresponding author.
