# Supplementary material for: Effects of mesenchymal stromal cells and human recombinant Nerve Growth Factor delivered by bioengineered human corneal lenticule on an innovative model of diabetic retinopathy
Source: Front Endocrinol (Lausanne). 2024 Oct 15;15:1462043. doi: 10.3389/fendo.2024.1462043 (PMC11518713; doi:10.3389/fendo.2024.1462043)
Supplement: Supplementary file 1 [file DataSheet1.docx]

Supplementary Material

## Supplementary Figures

**
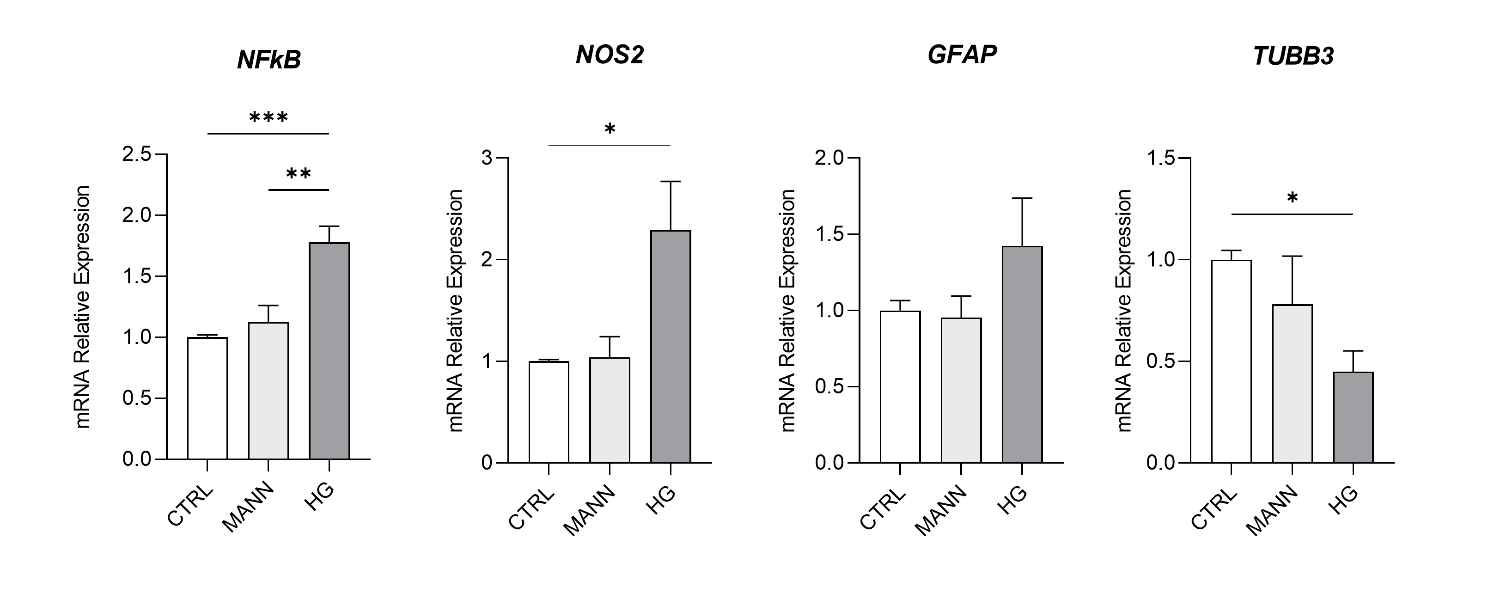
**

**Supplementary Figure 1.** Mannitol-treated porcine neuroretinal explants *ex vivo*. mRNA expression levels of inflammatory/oxidative (NFkB, NOS2 and GFAP) and structural (TUBB3) markers in porcine neuroretinal explants cultured for 4 days in the presence or absence of HG (25 mM) or Mannitol (25mM). Results are shown as the mean ± error standard (SEM) (n ≥ 5); *p<0.05; **p<0.01; *** p<0.001.
